# Salinity as a barrier for ship hull-related dispersal and invasiveness of dreissenid and mytilid bivalves

**DOI:** 10.1007/s00227-016-2926-7

**Published:** 2016-06-09

**Authors:** Marinus van der Gaag, Gerard van der Velde, Sander Wijnhoven, Rob S. E. W. Leuven

**Affiliations:** Department of Animal Ecology and Physiology, Institute for Water and Wetland Research, Radboud University Nijmegen, Heyendaalseweg 135, 6525 AJ Nijmegen, The Netherlands; Naturalis Biodiversity Center, P.O. 9517, 2300 RA Leiden, The Netherlands; Ecoauthor – Scientific Writing and Ecological Expertise, Leeuwerikhof 16, 4451 CW Heinkenszand, The Netherlands; NIOZ Royal Netherlands Institute for Sea Research, Utrecht University, P.O. Box 140, 4400 AC Yerseke, The Netherlands; Department of Environmental Sciences, Institute for Water and Wetland Research, Radboud University Nijmegen, Heyendaalseweg 135, 6525 AJ Nijmegen, The Netherlands; Netherlands Centre of Expertise for Exotic Species (NEC-E), Heyendaalseweg 135, 6525 AJ Nijmegen, The Netherlands

## Abstract

The benthic stages of Dreissenidae and Mytilidae may be dispersed over long distances while attached to ship hulls. Alternatively, larvae may be transported by water currents and in the ballast and bilge water of ships and vessels. To gain insight into dispersal potential and habitat suitability, survival of the benthic stages of two invasive dreissenid species (*Dreissena polymorpha* and *Mytilopsis leucophaeata*) and one mytilid species (*Mytilus edulis*) chosen based on their occurrence in fresh, brackish and sea water, respectively, were tested in relation to salinity. They were exposed to various salinities in mesocosms during three long-term experiments at outdoor temperatures. Mussel survival was studied without prior acclimation, reflecting conditions experienced when attached to ship hulls while travelling along a salinity gradient from fresh or brackish water to sea water, or vice versa. Initially, mussels react to salinity shock by temporarily closing their valves, suspending ventilation and feeding. However, this cannot be maintained for long periods and adaptation to higher salinity must eventually occur. Bivalve survival was monitored till the last specimen of a test cohort died. The results of the experiments allowed us to distinguish favorable (f.: high tolerance) and unfavorable (u.: no or low tolerance) salinity ranges in practical salinity units (PSU) for each species, viz. for *D. polymorpha* 0.2–6.0 PSU (f.), 7.0–30.0 PSU (u.), for *M. leucophaeata* 0.2–17.5 PSU (f.), 20.0–30.0 PSU (u.) and for *M. edulis* 10.5–36.0 PSU (f.), 0.2–9.0 and 40 PSU (u.). At the unfavorable salinities, all mussels died within 14 days of initial exposure with the exception of *M. edulis* (23–30 days). The maximum duration of survival of single specimens of *D. polymorpha* was 318 days at a salinity of 3.2 PSU, of *M. leucophaeata* 781 days at 15.0 PSU and of *M. edulis* 1052 days at 15.0 PSU. The number of days survived was compared with the duration of actual ship voyages to estimate the real world survival potentials of species dependent of salinity changes, travel distances and durations. The conclusion is that salinity shocks during the trip were survived within the favorable salinity range but that the species tolerate only for a few weeks the unfavorable salinity range. This functions as a barrier for dispersal. However, at faster and more frequent shipping in the future salinity can become no longer very important as a dispersal barrier.

## Introduction

Dispersal enables species to colonize suitable habitats in new areas and escape potential deteriorating conditions in their present habitat (Cain et al. 2000; Holt 2003; Lester et al. 2007). However, dispersal is often blocked by barriers. For the dispersal of aquatic species, land masses and mountain ridges are barriers. Also unfavorable water quality conditions such as too high or too low salinity levels for survival can prevent aquatic species to disperse and establish. Thus, sea straits and oceans can act as barriers for long-distance dispersal of freshwater species while rivers can be barriers for marine species when they are not tolerant for fresh water. These barriers are nowadays partly lifted by the high frequency and speed of seagoing ships and river vessels by which the chances for hitchhiking invasive species to survive the trip are very much increased as the period of exposure to unfavorable conditions decreased. A number of species of Dreissenidae and Mytilidae, which are known to spread in this way, are very successful invaders (Nalepa and Schloesser 1993; Van der Velde et al. 2010a; Nalepa and Schloesser 2013; Matthews et al. 2014).

Salinity and water temperature influence the survival, growth, activity and physiology of these bivalves (Bayne 1976; Gosling 1992a; Jansen 2009). These factors are therefore important for dispersal and establishment and determine the biogeographic distribution of these bivalve species (Kinne 1971; Schneider 2008; Lockwood and Somero 2011). Adaptive potential of species colonizing new sites may play an additional role in the range extension and invasiveness of several bivalve species. Fluctuating salinities such as those in estuaries give rise to a smaller range in salinity tolerance than stable salinities (Strayer and Smith 1993; Kilgour et al. 1994; Walton 1996; Orlova et al. 1998; Wilcox and Dietz 1998), while acclimation leads to a wider salinity tolerance range as also demonstrated by laboratory tolerance experiments using gradual or stepwise changes in salinity (Kilgour et al. 1994; Fong et al. 1995; Wright et al. 1996; Orlova et al. 1998; Wilcox and Dietz 1998).

Benthic Dreissenidae and Mytilidae stages can be transported when attached by their byssus threads to ship hulls during shipping and may even be transported overland for a limited period. Larvae and possibly benthic stages may disperse in ballast and bilge water. Rapid, long-range dispersal of benthic stages attached to ship hulls and the discharge of larvae in ballast and bilge water may result in either exposure to rapidly changing salinity gradients or sudden changes in salinity. This highlights the importance of deriving salinity tolerances which will increase understanding of the dispersal and establishment capacities of these invasive bivalve species. Salinity tolerances may be used to identify possible dispersal vectors based on survival chances.

In the present study, the salinity tolerance of the benthic stage of Conrad’s false mussel or dark false mussel, *Mytilopsis leucophaeata* (Conrad, 1831), was compared with that of the zebra mussel, *Dreissena polymorpha polymorpha* (Pallas, 1771) (further referred to as *D.polymorpha*) (both Dreissenidae), and the blue mussel, *Mytilus edulis edulis* L., 1758 (further referred to as *M. edulis*) (Mytilidae). No data on other related ‘subspecies’ or ‘species’ from the Baltic Sea, Caspian Sea, Aral Sea and Mediterranean Sea were included in this analysis.

*Dreissena polymorpha* is a freshwater species originating from the Ponto-Caspian area which has invaded most parts of Europe and large areas of North America (Van der Velde et al. 2010b; Benson 2014). It occurs in temperate and subtropical regions (Van der Velde et al. 2010b). *M. leucophaeata* is a brackish water species of North American origin which invaded Europe (Zhulidov et al. 2015). This species occurs mainly in tropical to subtropical and warm-temperate regions (Marelli and Gray 1983; Van der Velde et al. 2010b). *M. edulis* occurs in temperate regions and is native to the Atlantic coasts of Europe and North America (Gosling 1992b). If global distribution is taken into account, it is expected that these species differ in tolerance to various salinities in combination with water temperature, factors that may determine their invasion potentials.

Experiments using outdoor tanks (mesocosms) were undertaken to gain a better insight into these species ship transport-related dispersal and establishment potentials as indicated by salinity tolerance. Mussel survival was studied without prior acclimation, reflecting conditions experienced when attached to ship hulls while travelling along a salinity gradient or during a sudden shock from fresh or brackish water to sea water, or vice versa. In this way, favorable and unfavorable salinity survival ranges could be distinguished. Unfavorable salinities are supposed to function as barriers for dispersal. We tested this for a freshwater, brackish water and marine species to find out how far these species differ with respect to salinity as a barrier.

## Materials and methods

### Sampling sites

**Fig. 1:**
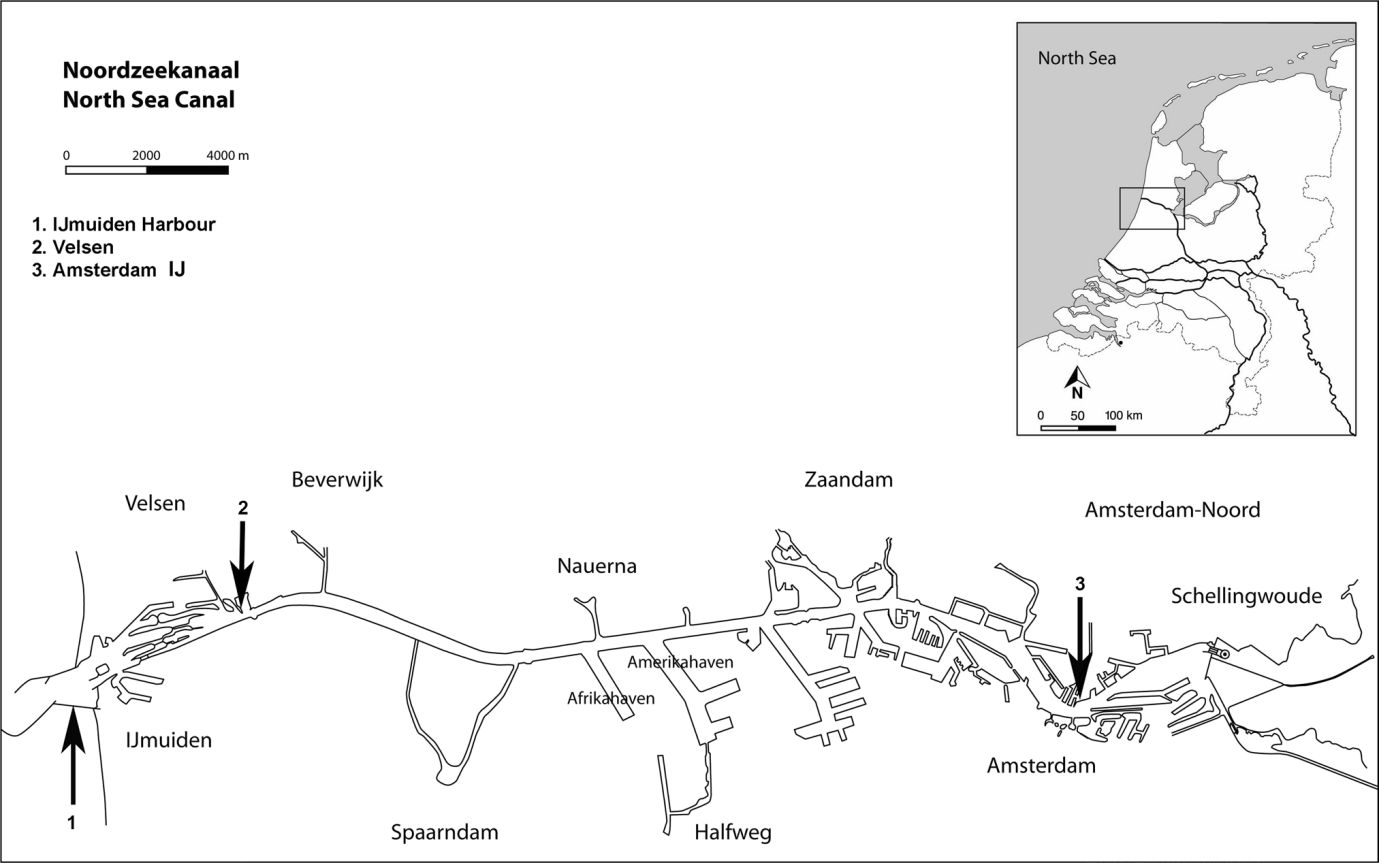
Map of the Noordzeekanaal (North Sea Canal) in the Netherlands with sampling sites: *1*: *Mytilus edulis*; *2*: *Mytilopsis leucophaeata*; *3*: *Dreissena polymorpha*

Mussels were collected from the North Sea Canal (Noordzeekanaal) at sampling locations featuring different salinities. The North Sea Canal connects the harbors of Amsterdam with the North Sea at IJmuiden, the Netherlands (Fig. 1). It features a salinity gradient that occurs due to the intrusion of sea water from the North Sea, the discharge of fresh water from the Rhine River and rain water from the Amsterdam harbors via the Amsterdam–Rhine Canal and Lake IJ. Salinity, expressed as practical salinity units (PSU), was measured with a salinity meter (YSI model 33 S-C-T) at all sampling sites. *M. edulis* was collected from the North Sea, on the outside of sluices near Velsen (salinity 17 PSU), *M. leucophaeata* was collected inside the sluices in the North Sea Canal (salinity 6 PSU) and *D. polymorpha* was collected from Lake IJ opposite Amsterdam’s central railway station (salinity 1.5 PSU) (Fig. 1).

### Experimental set-up for tolerance studies

The transfer of mussel specimens from their natural habitat to mesocosms containing water of different salinities means that mussels first have to survive the initial shock of altered salinity and subsequently adapt to the new salinity. This simulates the same shock experienced when ballast water exchange is used to eradicate mussels (Ellis and MacIsaac 2009). Mussels are capable of surviving salinity changes by closing their valves for a number of days with only short, intermittent opening periods that maintains the osmotic concentration in their mantle fluid (Bayne et al. 1976; Davenport 1979; Aunaas et al. 1988). If mussels survived this initial shock period, their survival at various stable salinities was studied until 100 % mortality occurred. In this way, data on long-term survival were obtained to assess habitat suitability for population establishment with respect to salinity.

Three long-term experiments with *D. polymorpha*, *M. leucophaeata* and *M. edulis* were performed in the period 1991–1995 on the campus of the Radboud University in Nijmegen (Table 1). Twelve outdoor concrete tanks (80 × 150 cm, height 60 cm) served as mesocosms and were buried in the ground (depth 50 cm). The mesocosm inner walls were lined with PVC. The ground between the mesocosms was paved with concrete slabs to prevent plant growth. The mesocosms were covered with chicken wire to prevent leaf litter and terrestrial animals falling into the tanks.

**Table 1:** Start and end dates (100 % mortality) of the three experiments with *Dreissena polymorpha,* *Mytilopsis leucophaeata* and *Mytilus edulis*

Experiment	Species	Start	End
1	*D. polymorpha*	September 3, 1991	July 7, 1992
	*M. leucophaeata*	August 25, 1991	July 15, 1992
2	*D. polymorpha*	October 13, 1992	July 6, 1993
	*M. leucophaeata*	October 2, 1992	November 30, 1994
	*M. edulis*	September 29, 1992	July 27, 1995
3	*D. polymorpha*	May 4, 1993	November 2, 1993
	*M. leucophaeata*	April 26, 1993	March 6, 1995

Mussel survival was equated to tolerance and studied in a salinity gradient. The salinity gradient was created by varying salinity concentration over the series of mesocosms and prepared by mixing water collected outside (salinity 17 PSU) and inside the sluices (salinity 6) of the North Sea Canal with fresh water collected near Nijmegen from the Waal River, the main distributary of the Rhine River in the Netherlands. The water was not filtered before use. Salinities higher than 17 PSU were produced by mixing sea water from outside the sluices with 10 % river water and Mediterranean Sea salt produced for sea aquaria use.

During the experiments, salinity was checked weekly using an YSI model 33 S-C-T meter. Salinity levels were kept stable by adding sea salt after periods of rain, or tap water after periods of evaporation at high temperatures. The measured deviation from the initial salinity was always less than 10 %. The water temperature was measured weekly with a mercury thermometer. In each mesocosm, a small air compressor and a bubble stone maintained the oxygen content. The air bubbles caused constant mixing of the water in the mesocosm.

The mussels were stocked in nylon nettings of size 30 × 15 cm, mesh size 1 mm; in most cases, 24 mussels belonging to several size classes were added per netting (3 specimens per size class). This was done to ensure that all mussels were present at the same depth and are exposed in this way to similar conditions and for a practical reason, viz. that all mussels could easily be taken out of the water and studied. Each nylon netting was marked with a number and was attached to the chicken wire covering with a rope located in the center of the mesocosm and allowed to hang freely in the water at a depth of approximately 25 cm. Depending on the number of test species in the mesocosm (i.e., one, two or three species), two, four or six nets hung in a mesocosm, respectively (Table 1). The lengths of all mussel shells were measured before they were used in the experiment with a vernier caliper that has an accuracy of 0.1 mm. The mussels were not marked individually. To mimic ship transport, the mussels were not acclimated before they were exposed to the salinities in the mesocosms and thus were added after collection in the field directly to the mesocosms. No food was added to the mesocosms, so that mussels were dependent on sources of nutrition initially present and spontaneously developed in the water.

In the first experiment that occurred in 1991–1992 (Table 1), the salinity gradient consisted of the 12 mesocosms containing salinities of 0.5, 1.7, 3.2, 6.0, 7.0, 8.5, 10.0, 12.0, 14.0, 17.0, 20.0 and 30.0 PSU after mixing. In the second experiment of 1992–1995 (Table 1), the salinity gradient consisted of 12 mesocosms with salinities of 0.2, 2.0, 4.0, 7.5, 9.0, 10.5, 13.0, 15.0, 17.5, 30.0, 36.0 and 40 PSU after mixing, to which all three bivalve species were exposed, except for 36 PSU which was not used for *D. polymorpha* and 40 which was only used for *M. edulis*. In the third experiment occurring in 1993–1995 (Table 1), the salinity gradient consisted of 11 mesocosms with salinities of 0.2, 2.0, 4.0, 6.0, 7.5, 9.0, 10.5, 13.0, 15.0, 17.5 and 30.0 PSU after mixing to which *D. polymorpha* and *M. leucophaeata* were exposed. The nettings were opened every week for inspection. Individuals that were still alive were counted, put back into the nettings and hung back in the mesocosms. Empty shells and dead mussels identified by their open shells were removed and counted, their shell lengths measured and the date when death was established was recorded. Subsequently, water temperature and salinity were measured. The salinity of the water in each mesocosm was adjusted to the initial level when necessary as described previously. Analyses were performed using length of survival, water temperature and numbers of dead and living mussels to calculate survival percentages. The influence of mussel size on species survival capacity was also analyzed (see multivariate analysis).

### Mussels and size classes

Three long-term experiments were performed with the three bivalve species (Table 1). During the three experiments, the selected shell length classes of *D. polymorpha* were 4–5, 6–7, 8–9, 10–11, 12–13, 14–15, 16–17 and 18–26 mm. In the experimental periods, 48 individuals were added per mesocosm, distributed over two nettings. During the three experiments, the selected shell length classes for *M. leucophaeata* were 4–5, 6–7, 8–9, 10–11, 12–13, 14–15, 16–17 and 18–23 mm. In the experimental periods, 48 individuals were added per mesocosm, distributed over two nettings.

During experiment two (1992–1995), also nine size classes were selected for *M. edulis* that, for the most part, differed by 3 mm (4–7, 8–11, 12–15, 16–19, 20–23, 24–27, 28–31, 32–35 and 36–49 mm), resulting in a total of 54 *M. edulis* individuals per mesocosm distributed over two nettings.

### Multivariate statistics

To combine the results of all experiments and extract general patterns, principal component analyses (PCAs) were applied to identify relationships between patterns in survival of different mussel species and relations with environmental and treatments characteristics and patterns in longevity of different mussel species and relations with environmental and treatments characteristics. In the first case (PCA of survival data), percentages of specimens surviving (being alive) as recorded at regular intervals for each of the experimental batches of mussels are used as input data. The input measurements consist of dependent measurements in time for the same batches for which survival likely decreases in time (number of days after the start of the experiment: days), which can however be a stronger or less strong relation dependent of the species, and the salinity conditions. Moreover, using input data from different experiments allows to analyze the impact of factors like temperature and temperature history (indicated as temperature fluctuation: a summation of the temperature difference between an observed maximum and a minimum water temperature for each of the periods that the trend in temperature changes turns which equals the period of increase from winter to summer plus decrease from summer to winter, *etcetera* till mortality) as these differ between experimental years. In the second case (PCA of longevity data), individual specimen-specific input data, i.e., number of days specimens have survived in the experiment from start to mortality, are used as input data. Patterns among species and experimental conditions are also related to specimen-specific aspects as measured at the start (initial size of the mussels as classified into size groups) and the, for that specimen, end of the experiment, when the specimen appeared to have died (size at death as indicated as shell length, time of the year the specimen has died as indicated by the season). PCAs were used for the analyses as detrended component analysis (DCA) indicated short gradient length in the species data. Additionally, these indirect gradient analyses were used, as PCAs, as there was particular interest in determining which factors were most important in explaining the observed patterns in mussel survival and longevity. In order to make the three experimental periods comparable, the experimental salinity was classified using four salinity classes (fresh to oligohaline 0.2–4.0 PSU; low mesohaline 6.0–10.0 PSU; high mesohaline 12.0–17.5 PSU; polyhaline to mixoeuhaline 20.0–40.0 PSU) in accordance with the ‘Final resolution of the symposium on the classification of brackish waters’ (Battaglia 1959). The postmortem shell lengths were classified into eight to nine size classes (depending on the species) similar to the classes used at the start of each experiment, and the dates when death was established were classified according to season (spring March 20–June 20, summer June 21–September 22, autumn September 23–December 20 and winter December 21–March 19). All data were log-transformed according to *y* = log(*x* + 1) before analyses to account for zero values and reduce the impact of extreme values. Multivariate statistics were carried out using CANOCO for Windows v4.5 (Ter Braak and Smilauer 2002).

## Results

**Fig. 2:**
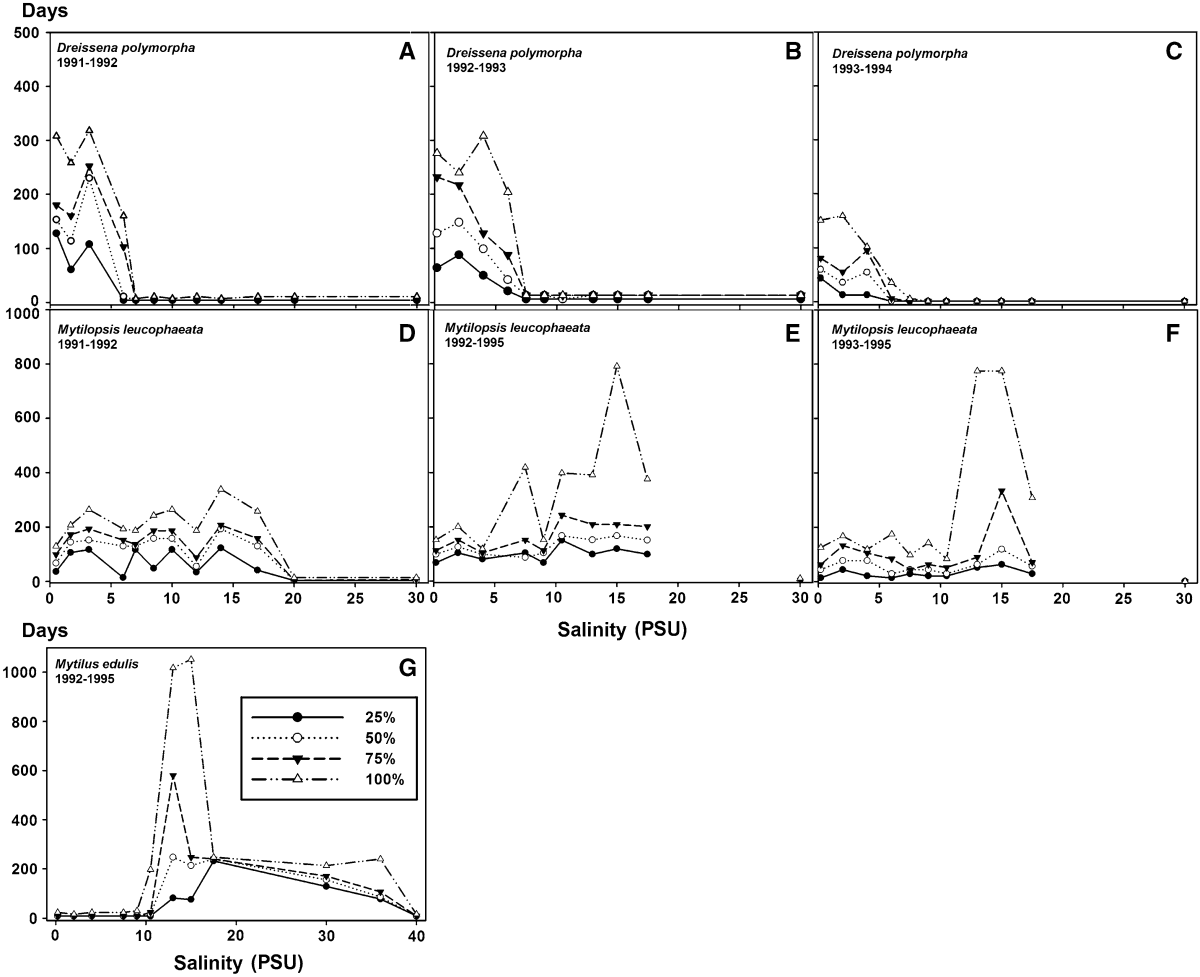
Mortality (%) and survival (maximum number of days) of *Dreissena polymorpha,* *Mytilopsis leucophaeata* and *Mytilus edulis* at various salinities during various experimental periods

During experiment one, *D. polymorpha* showed a high tolerance (100 % mortality in 318 days) within a salinity range of 0.5 to 3.2 PSU, a decreased tolerance (100 % mortality in 164 days) at salinity 6.0 PSU and a very low tolerance (100 % mortality in 11 days) at salinities of 7.0 PSU and higher. *D. polymorpha* showed the highest tolerance at a salinity of 0.5–3.2 PSU. During experiment two, *D. polymorpha* showed a high tolerance (100 % mortality in 308 days) at salinities between 0.2 and 6.0 PSU and the highest tolerance at salinities below 4.0 PSU. During experiment three, *D. polymorpha* showed a high tolerance (100 % mortality in 159 days) at the salinities between 0.2 and 4.0 PSU, a lower tolerance (100 % mortality in 36 days) at a salinity of 6.0 PSU, a mortality of 100 % in 13 days at a salinity of 7.5 PSU and a very low tolerance (100 % mortality in 6 days) at salinities above 7.5 PSU. *D. polymorpha* showed the highest tolerance at a salinity of 2.0 PSU (Fig. 2).

During experiment one, *M. leucophaeata* showed a high tolerance (100 % mortality in 332 days) within a salinity range of 0.5 to 17.0 PSU and low tolerance (100 % mortality in 7 days) at salinities of 20.0 PSU and higher (Fig. 2). *M. leucophaeata* showed the highest tolerance at a salinity of 14.0 PSU. During experiment two, *M. leucophaeata* showed a high tolerance (100 % in 781 days) at salinities between 0.2 and 17.5 PSU and the highest tolerance at a salinity of 15.0 PSU. During experiment three, *M. leucophaeata* showed a high tolerance at salinities ranging from 0.2 to 17.5 PSU with the highest tolerance at a salinity of 15.0 PSU (100 % in 655 days). *M. leucophaeata* showed the highest tolerance at a salinity of 13.0–15.0 PSU (Fig. 2).

During experiment two, *M. edulis* showed a high tolerance (100 % mortality in 1052 days) at salinities between 10.5 and 36.0 PSU. *M. edulis* showed the highest tolerance at a salinity of 15.0 PSU (Fig. 2).

From the results of these experiments, favorable (high tolerance) and unfavorable (low and no tolerance) salinity ranges could be derived for the three species, viz. for *D. polymorpha*: 0.2–6.0 and 7.0–30 PSU, for *M. leucophaeata*: 0.2–17.5 and 20.0–30.0 PSU and for *M. edulis*: 10.5–36.0 and 0.2–9.0 and 40 PSU, respectively.

**Fig. 3:**
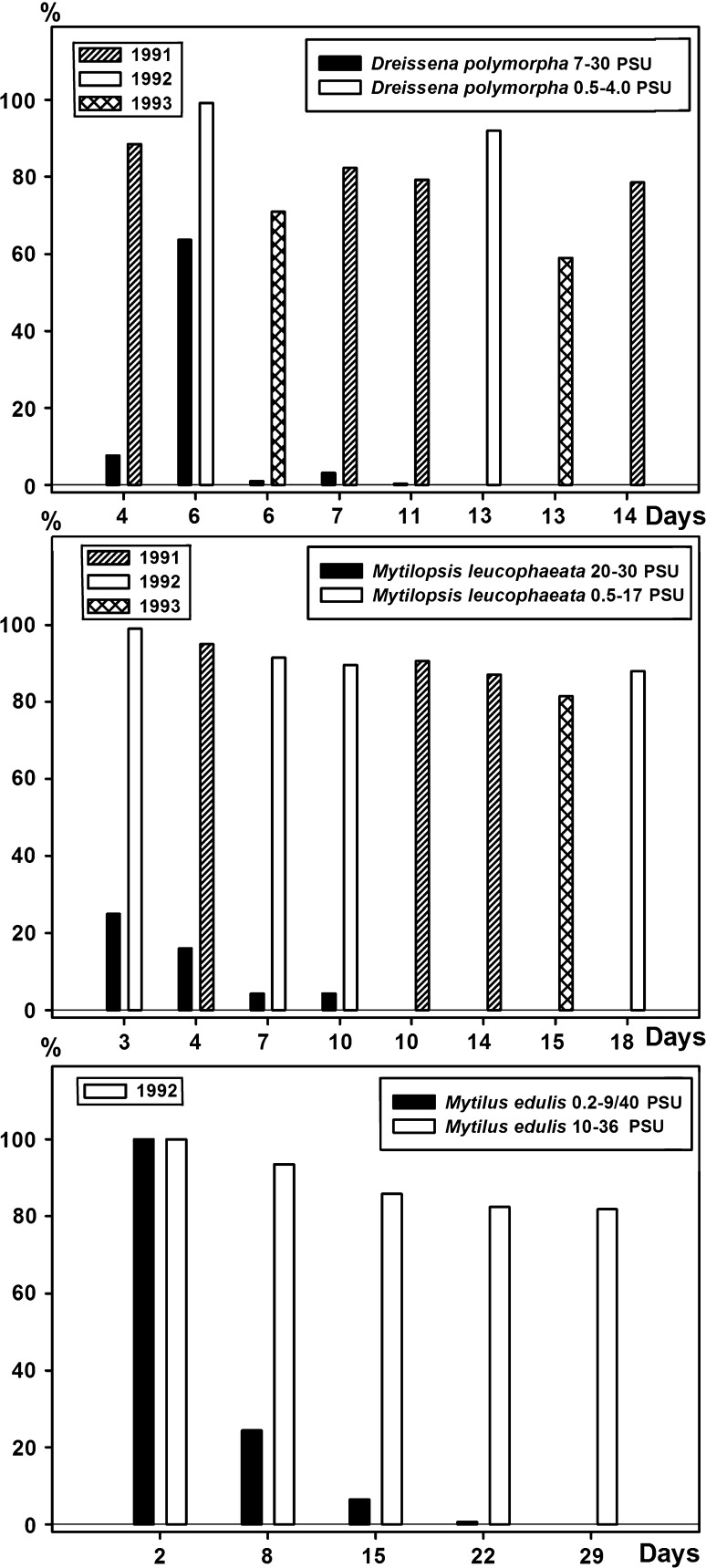
Salinity shock tolerance of *Dreissena polymorpha*, *Mytilopsis leucophaeata* and *Mytilus edulis* expressed as a survival percentage at days following transfer to low-tolerance and high-tolerance experimental salinities in mesocosms based on all experiments

Mortality as a result of salinity shock occurring directly after the introduction of specimens in the mesocosms was low at the previously defined, favorable salinities but high at unfavorable salinities for all three species. Resistance of the species at unfavorable salinities differed. This was in the case of both dreissenid species generally not longer than 15 days. A longer resistance was observed for *M. edulis* (29 days). The highest mortality was observed in the first week of exposure for all species (Fig. 3).

Shell growth was nearly negligible for all species at favorable salinity ranges during all experiments. Based on shell length measurements recorded at the start of the 1992–1995 period, and on the shell lengths of dead mussels, *D. polymorpha* shells grew by an average of 0.04–0.16 mm (0.2–6.0 PSU), *M. leucophaeata* by an average of 0.11–0.25 mm (0.2–17.5 PSU) and *M. edulis* by an average of 0.91 mm (10.5–36.0 PSU) over the whole experimental period till their death.

**Fig. 4:**
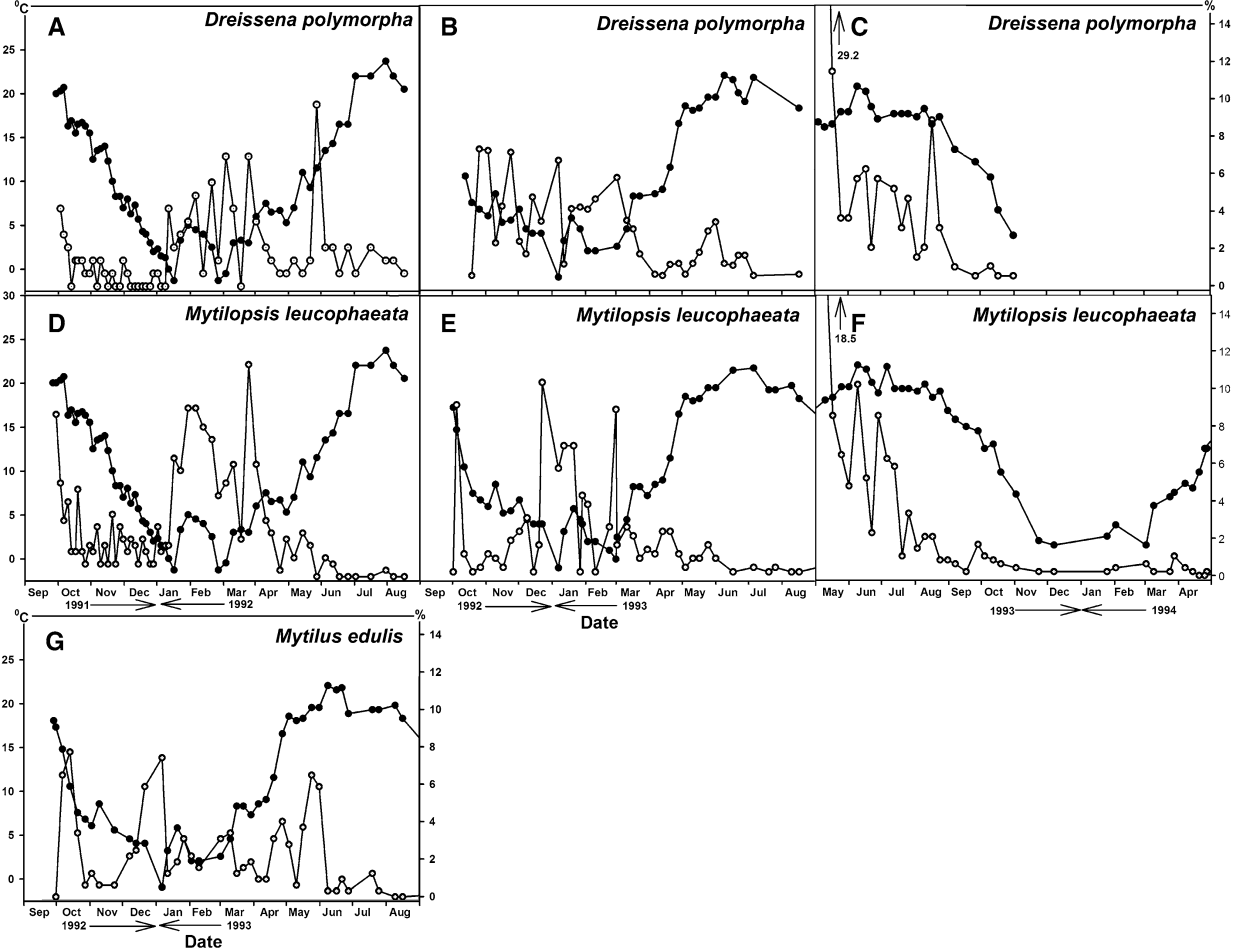
Water temperature (*closed circles*) and mortality (*open circles*) of *Dreissena polymorpha* at salinity 0.2–6.0 PSU (1991–1992: N = 192; 1992–1995: N = 170; 1993–1994; N = 192) (**a**, **b**, **c**), *Mytilopsis leucophaeata* at salinity 0.2–17.5 PSU (1991–1992: N = 480; 1992–1995: N = 415; 1993–1994: N = 480) (**d**, **e**, **f**) and *Mytilus edulis* at salinity 13.0–36.0 (1992–1995: N = 270) (**g**). The graphs of 1992–1995 of *D. polymorpha* and *M. leucophaeata* were not continued after 1993 as *D. polymorpha* was already extinct and the numbers of *M. leucophaeata* strongly reduced

At favorable salinities, mortality of *M. leucophaeata*, *D. polymorpha* and *M. edulis* was high in winter when the water temperature decreased to ≤5 °C (Fig. 4). In the cold period that occurred beween December 2, 1991, and March 30, 1992, 86 of 192 *D. polymorpha* individuals (44.8 %) and 277 of 480 *M. leucophaeata* individuals (57.7 %) died. In the cold period that occurred between December 9, 1992, and March 11, 1993, 237 of 415 *M. leucophaeata* individuals (57.1 %), 42 of 129 *D. polymorpha* individuals (32.6 %) and 107 of 270 *M. edulis* individuals (39.6 %) died. Following a reduction in water temperature to below 0 °C on the 7th of January, 1993, a high mortality peak was observed for all species (Fig. 4). The 1993–1995 experimental period commenced earlier in the year than the other experiments, resulting in high mortality during the summer period. Therefore, mortality percentages at low temperatures could not be calculated for this experiment, as no *D. polymorpha* was left anymore and due to the very low remaining numbers of *M. leucophaeata* in the winter period (Fig. 4).

**Fig. 5:**
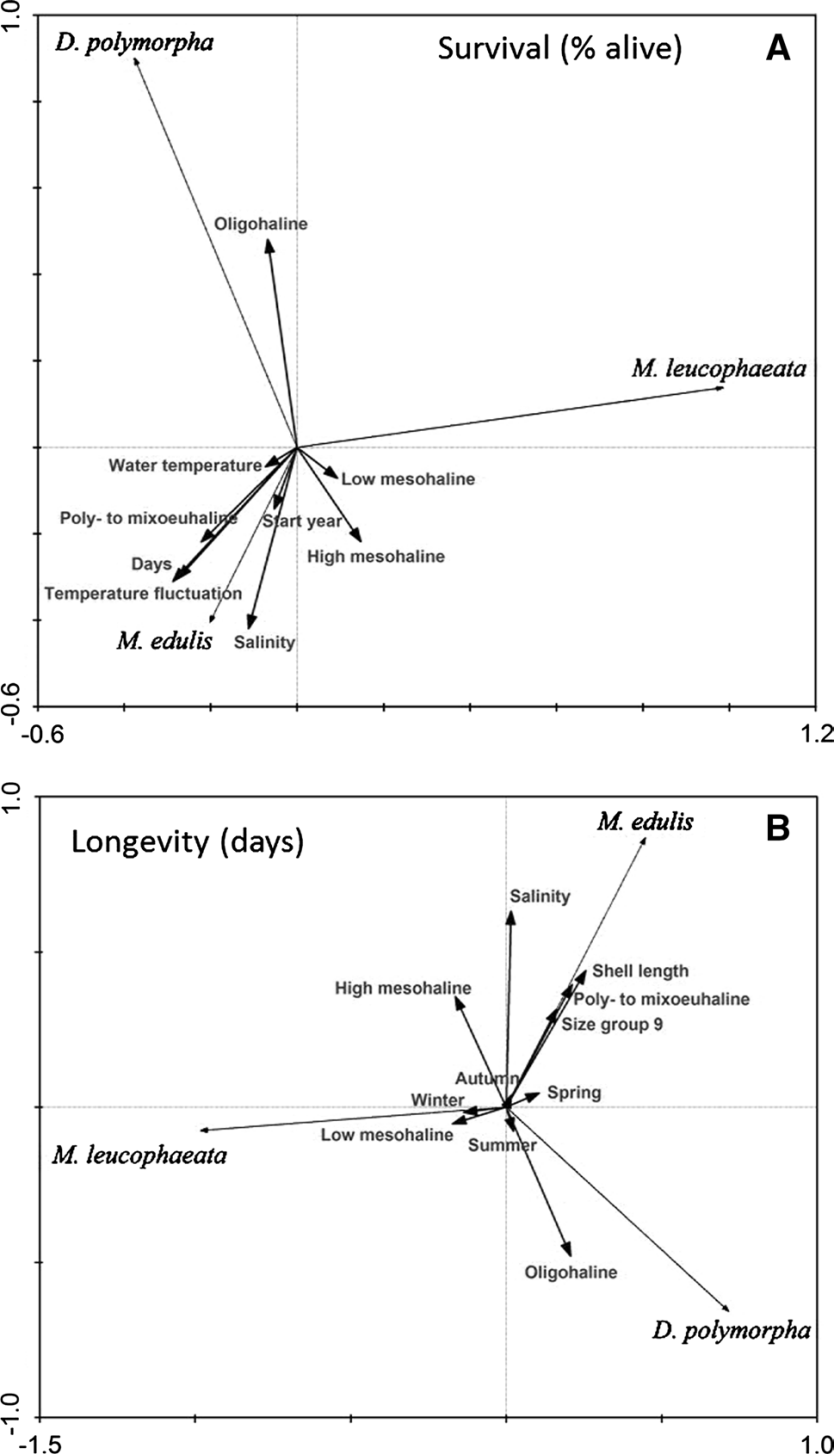
**a** Ordination (PCA) of mussel survival in percentages of batches of different species and size ranges during three sets of experiments with different experimental salinities and varying environmental conditions. **b** Ordination (PCA) of mussel longevity in days of individual mussel specimens during three sets of experiments with different experimental salinities and varying environmental variables. Size group 9 consisted of specimens with a shell length between 36 and 49 mm which is solely *Mytilus edulis*. All other size groups and the year of the start of the experiments were excluded from the graph as their correlations with species were only minor

The PCA results show that *D. polymorpha* suffered the least mortality at oligohaline conditions whereas mortality is particularly high at salinities above 12.0 (high mesohaline) (Fig. 5a; Table 2). The projection of the number of days (age of specimens when mortality is measured), the water temperature and the temperature fluctuation, on the *D*. *polymorpha* arrow, is very short. This indicates that there were *D. polymorpha* individuals that died very quickly, and individuals that survived much longer, which is related to salinity and not water temperature. The survival of *M. leucophaeata*, however, appeared not to depend on lower salinities (oligohaline to high mesohaline), but mortality increased with higher salinities (poly- to mixoeuhaline). This species typically on average survived shorter than *M. edulis*, so that survival is particularly related to shorter experimental duration time (i.e., days). This automatically means that also temperature fluctuation was lower for *M. leucophaeata* than for *M. edulis* for who specimens on average went through several seasonal temperature changes before they died. *M. edulis* shows low mortality when exposed to higher salinities, typically above 20.0, for a long duration. At lower salinities, *M. edulis* survival is similar to that of *M. leucophaeata* (Fig. 5b). The average longevity of specimens of *M. leucophaeata* was slightly higher at low mesohaline conditions than at high salinities (poly- to mixoeuhaline). The relatively short arrows relating to season indicate that salinity was the most important factor determining the longevity of mussels, and not season-related aspects like water temperature or health status impacting the animals. Generally, size did not influence survival in any species. Only larger-sized *M. edulis* specimens (36 and 49 mm shell length, size group 9 in Fig. 5a) had the capacity to survive longer during the experiments compared to their smaller counterparts. Larger-sized *M. edulis* specimens may therefore display greater resistance against suboptimal salinity.

**Table 2:** Results of principle component analyses (PCAs) of bivalve species survival (Fig. 5a) and bivalve species longevity (Fig. 5b) related to environmental conditions and specimen characteristics

	Axis 1	Axis 2
Summary statistics of first two canonical axes of Fig. 5a (mussel survival)		
Eigenvalues	0.594	0.256
Species–environment correlations	0.396	0.579
Cumulative percentage variance		
of species data	59.4	85.0
of species–environment relation	46.5	89.1
Correlation of environmental variables with canonical axes of Fig. 5a (mussel survival)		
Days	−0.2751	−0.3030
Water temperature	−0.0729	−0.0448
Salinity	−0.1118	−0.4198
Start year	−0.0527	−0.1421
Oligohaline	−0.0674	0.4814
Low mesohaline	0.0927	−0.0712
High mesohaline	0.1475	−0.2176
Poly- to mixoeuhaline	−0.2219	−0.2187
Temperature fluctuation	−0.2873	−0.3100
Summary statistics of first two canonical axes of Fig. 5b (mussel longevity)		
Eigenvalues	0.628	0.330
Species–environment correlations	0.589	0.825
Cumulative percentage variance		
of species data	62.8	95.9
of species–environment relation	47.6	96.7
Salinity	0.0149	0.6314
Shell length	0.2551	0.4399
Oligohaline	0.2071	−0.4798
Low mesohaline	−0.1729	−0.0534
High mesohaline	−0.1634	0.3561
Poly- to mixoeuhaline	0.2112	0.3944
Size group 9 (36–49 mm)	0.1623	0.3163
Winter	−0.1372	−0.0172
Spring	0.1057	0.0450
Summer	0.0230	−0.0775
Autumn	0.0169	0.0397

## Discussion

During our experiments, maximum temperature did not exceed 24 °C in the summer period. Additionally, in summer in the mesocosms, planktonic algae developed which served as food. During laboratory experiments, Chase and McMahon (1995) found that *D. polymorpha* appeared to be extremely tolerant to starvation, having a LT_50_ of 118 days and a LT_100_ of 143 days at 25 °C, and a LT_50_ of 352 days and a LT_100_ of 545 days at 15 °C. *D. polymorpha* individuals kept at 5 °C survived longer than 600 days without reaching 100 % mortality. Therefore, starvation was not considered to be an important factor contributing to mortality in our mesocosms.

Salinity shocks occurring in our experiments, that simulate conditions that occur during transport overseas and during ballast water exchange, could have reduced the salinity ranges to ranges more typical for estuaries than for brackish water lakes. Salinity shocks were observed to cause a rapid high mortality at the unfavorable salinity ranges imposed during our experiments in contrast to shocks within favorable ranges.

Mackie and Claudi (2010) present levels of infestation by *D. polymorpha* based on the literature data at different salinities in North American water bodies. They concluded that no potential for adult survival exists at salinities over 10 PSU, a moderate potential for nuisance infestations exist at salinities between 5 and 10 PSU and a high potential for massive infestations exists at salinities below 5 PSU. We found a low mortality of *D. polymorpha* in salinities ranging from 0.2 to 6.0 PSU and an optimal survival at salinities below 4 PSU. In 1991, the longest living *D. polymorpha* individual died after 318 days at a salinity of 3.2 PSU. In 1992, the last *D. polymorpha* individual died after 164 days at a salinity of 6.0 PSU. The results of our experiments are in agreement with the results of Mackie and Claudi (2010).

The benthic phase of *M. leucophaeata* can survive salinities as low as 0.1 and as high as 17.5 PSU (Zhulidov et al. 2015 and the literature discussed therein). Field transplantation experiments confirm that individuals can stay alive in fresh water for 4 months under winter conditions (Verhofstad et al. 2013). Our experiments also demonstrated a high tolerance of *M.leucophaeata* for fresh water. Complete mortality occurred in our mesocosms at a salinity of 20 PSU within two weeks. In Europe, this species most likely occurs in fresh water to high mesohaline water. There are records of *M. leucophaeata* in the freshwater parts of rivers, but these individuals are only present because individuals are regularly introduced via ships from brackish harbors (Steussloff 1939; Jaeckel 1962) as is evident from calcareous tube worms and brackish water bryozoans present on their shells which do not occur in fresh water (Kelleher et al. 1997, 1999). Mackie and Claudi (2010) present data on levels of infestation by *M. leucophaeata* at different salinities in North America. The authors observed that there is no potential for adult survival at salinities of <0.2 PSU or >30 PSU; a moderate potential for nuisance infestations at salinities between 2–4, 12–25 PSU; and a high potential for massive infestations at salinities in the range of 5–12 PSU. Wolff (1969) notes that *M. leucophaeata* occurs in brackish waters only when fluctuations in salinity are slow and where there are no strong daily fluctuations.

Experiments on the salinity tolerance of *M. leucophaeata* were performed in the USA. Deaton et al. (1989) found that *M. leucophaeata* from Florida (USA) living in aquaria in deionized water had a high survival rate for 3 weeks after which a gradual decline occurred till day 80 by which time all mussels had died. In freshwater conditions (salinity of 0.2 or 0.4 PSU), half of the animals died during the same period. A reduced mortality rate was observed at salinities of 1.6 and 6.4 PSU, and a higher mortality rate was observed at salinities in the range of 12.8 to 19.2 PSU. At salinities above 19.2 PSU, the animals died very rapidly. The experiments of Deaton et al. (1989) reveal that the optimal salinity range is 6.4–12.8 PSU and high survival occurs in the range of 1.6–19.2 PSU. In laboratory experiments with a duration of 42 days, Castagna and Chanley (1973) found that in salinities of 0–30.0 PSU, 80–100 % of the *M. leucophaeata* survived and produced byssus threads (the mussels were collected at a salinity of 7 and kept several weeks at a salinity of about 17.5 PSU prior to experimentation). No mortality was associated with reciprocal transfers between salinities of 2.5 and 27.5 PSU. One week after the transfer, the mussels were attached and filtering as usual. Salinity tolerance limits as determined from natural distribution and laboratory experiments were below 12 PSU in nature and 0 as minimum in laboratory experiments according to Castagna and Chanley (1973). In our long-term experiments, *M. leucophaeata* displayed a low mortality in the salinity range of 0.2–17.5 PSU and survived optimally at a salinity of 15 PSU.

In laboratory experiments with a duration time of 40 days, Almada-Villela (1984) established a threshold for shell growth of *M. edulis* close to a salinity of 12.8 PSU. The mussels were kept for 1 week at a salinity of 32 PSU before they were used in the gradient experiment. Experiments with a salinity of 9.6 PSU or lower were not possible because all mussels died within 10 days at these salinities. Wolff (1969) stated that the lower salinity limit of regular occurrence of *M. edulis* in Dutch estuaries is 8–10 PSU. In our experiments, *M. edulis* mortality was low at salinities ranging from 10.5 to 36 PSU and optimal survival occurred at a salinity of 15.0 PSU. A much longer survival time of 184 days for 25 % of the mussels and a survival time of 1052 days for the longest living individual of *M. edulis* were observed under these conditions. Where salinity was 9.0 PSU or lower, 92.2 % died within 15 days and all mussels (collected at a salinity of 17) died within 30 days. The *M. edulis* mussels used in our experiments were collected in the harbor of IJmuiden just outside the sluices of the North Sea Canal. Brackish water that enters during the opening of sluices that allow ship passages may have allowed these mussels to adapt to lower salinities (17 PSU) than those occurring in ocean water (salinity 35 PSU). This may be a reason for the low optimum salinity value for survival of *M. edulis* observed in our experiments.

Survival of attached mussels on the hulls of seagoing ships is dependent on their tolerance to sea water for which the three species tested showed clear differences. Survival when attached to ships is limited in sea water for *M. leucophaeata* and *D. polymorpha* and in low brackish- and fresh water for *M. edulis*. Survival is dependent on the tolerance of salinity levels, fluctuations, shocks and time exposed to the various conditions (e.g., seasonal) and the time that a ship is amenable to mussel colonization. Their survival is also dependent on the duration of the trip from one harbor to another.

From our tolerance experiments, it can be concluded that *M. edulis* may be easily transported by seagoing ships from one continent to another without significant mortality. However, *D. polymorpha* showed a low tolerance for such a salinity shock and 100 % mortality will occur within 11–13 days at salinities higher than 6 PSU, depending on the season. *M. leucophaeata* is more tolerant for higher salinities but, similarly to *D. polymorpha*, cannot survive in sea water for longer than 10 days (100 % mortality). This means that the dispersal of both dreissenids through attachment to seagoing ships is unlikely to occur. Survival of these species may occur during short, fast sea trips, where the right freshwater or brackish water conditions exist in the harbors where the ship berths. Dispersal is also dependent on the period of time allowed for attachment of these species to ship hulls during berthing.

The benthic mussel stage can be dispersed when attached to ship hulls. Mussel species surviving in fresh water can be dispersed via ship transport in rivers and canals. With increased vessel speed and vessel traffic and a complete network of rivers and canals over continental Europe (Leuven et al. 2009; Bij de Vaate et al. 2013), these species have the opportunity to settle everywhere where more or less stable brackish water conditions occur. However, in the Mediterranean, there is still a wide distribution gap of *M. leucophaeata* between the southern coast of France and the localities in the Black Sea (Zhulidov et al. 2015). This part of Europe including Italy, the Balkan states and Greece, falls outside the European canal-river network and *M. leucophaeata* can only colonize harbors in these regions via seagoing vessels. Stable brackish water gradients only occur locally along the sea coast. Therefore, each isolated population in a colonized brackish water body has to function as a stepping stone for further dispersal.

In the case of *M. edulis*, fresh water forms a barrier. The species can tolerate freshwater–oligohaline conditions for 17 days. However, the species is able to survive at salinities of more than 10.5 in brackish water harbors. Some figures on the duration of voyages for freshwater-going vessels that travel from harbors via European rivers and connecting canals could be found on the internet (Table 3). These durations mean that attached *M. edulis* has a low chance of survival in contrast to *D. polymorpha* and *M. leucophaeata* if transported via the river network from the North Sea to the Black Sea and vice versa. To get an impression of travel times of seagoing ships, data found on the internet in 2015 were also summarized in Table 3.

**Table 3:** Travel time of seagoing and river-going ships found on internet in 2015

Harbors	Time (days)	Ship	References
Hoek van Holland–Harwich	0.23	Ferry	www.stenaline.nl
Antwerpen–Hanko (Finland)–Rauma (Finland)–Antwerpen	8	Containership 205 × 26 m, 17,611 tdw	www.cargoshipcruises.nl
Rotterdam–Reydarfjodur (Iceland)–Hull (GB)–Rotterdam	14	Containership 145 × 18 m, 10,450 tdw	www.cargoshipcruises.nl
IJmuiden–Cleveland (USA)	12–14	Containership 200 × 25 m 35,000 tdw	www.cargoshipcruises.nl
IJmuiden–Burns Harbor (Chicago, USA)	21	Containership 200 × 25 m 35,000 tdw	www.cargoshipcruises.nl
Rotterdam–Suez Canal–Hongkong (China)–Hamburg–Rotterdam	70	Containership 334 × 43 m 100,864 tdw	www.cargoshipcruises.nl
Antwerpen–Gebze (Turkey)– Amberli (Istanbul)–Gemlik (Turkey)–Antwerpen	21	Containership 294 × 32 m, 63,428 tdw	www.cma-cgm.com
Arnhem (NL)–Passau (Germany)	14	Cruise ship	www.vikingrivercruises.com
Passau–Tulcea (Romania)	7	Cruise ship	www.vikingrivercruises.com
Amsterdam–Bucharesti (Romania)	23	Cruise ship	www.vikingrivercruises.com

*tdw* total dead weight

The dispersal success of the three studied species by attachment to vessels is not only dependent on their tolerance, but also on their densities on the ship hull which is dependent on the time allowed to settle when a ship is berthed, their propagule pressure, and the conditions at the received area with respect to growth and reproduction (Van der Velde et al. 2006).

Alternatively, *D. polymorpha*, *M. leucophaeata* and *M. edulis* can be dispersed overseas by transport as larvae within ballast water, and in rivers as bilge water in ships and vessels. According to the results of laboratory experiments carried out by Kilgour et al. (1994), salinity limits for adult and veliger larvae of *D. polymorpha* were > 4–8 PSU (at acclimation) and 4.5 PSU (acclimated and non-acclimated), respectively, but post-veliger larvae were more sensitive and determine perhaps the distribution of *D. polymorpha* in brackish water systems. Development of settled pediveligers to juveniles of *D. polymorpha* occurred up to a salinity of 6 PSU in laboratory experiments by Wright et al. (1996). Mackie and Claudi (2010) stated that little potential for larval development of *D. polymorpha* exists at a salinity of 8–10 PSU. An American experimental study by Siddall (1980) showed that *M. leucophaeata* larvae seem to have a much greater tolerance of high salinities compared to the benthic stage. Larvae can tolerate a salinity up to 32 PSU with normal development. However, based on the available literature, Mackie and Claudi (2010) concluded that there is little potential for larval development of *M. leucophaeata* at salinities of 0.2–2 and 25–30 PSU. A combined study of the effects of salinity and temperature on larvae of *M. edulis* by Brenko and Calabrese (1969) showed that survival of larvae is uniformly good at salinities of 15 to 40 PSU at temperatures from 5 to 20 °C but is reduced drastically at 25 °C, and partially at high (40 PSU) and low (20 PSU) salinities. Optimum growth occurs at 20 °C and salinities from 25 to 30 PSU.

## Conclusions

Salinity was found to be the most important factor determining the longevity of the benthic stage of *D. polymorpha*, *M. leucophaeata* and *M. edulis* in the outdoor mesocosms as opposed to season, shell length or water temperature. At the unfavorable salinities, all mussels died within 14 days of initial exposure with the exception of *M. edulis* (23–30 days). Within the favorable salinity ranges, the salinity change leads only to low mortality. The maximum duration of survival of single specimens of *D. polymorpha* was 318 days at a salinity of 3.2 PSU, of *M. leucophaeata* 781 days at a salinity of 15.0 PSU and of *M. edulis* 1052 days at a salinity of 15.0 PSU. At favorable salinity ranges, low temperatures caused mortality that was clearly higher for *M. leucophaeata* than for *D. polymorpha* and *M. edulis*. The found favorable salinity ranges correspond with salinity limits mentioned in the literature for estuaries where salinity shocks occur. Results of laboratory experiments from the literature were sometimes somewhat contradictory which could be caused by differences in acclimation, source populations, handling, experimental design and duration of the experiments. It can be concluded that the salinity tolerance of larvae as mentioned in the literature is more restricted or at most similar to those of the benthic stages in the case of *D. polymorpha* and *M. edulis*. This in contrast to *M. leucophaeata* for which the larvae seem to be most tolerant; i.e., even tolerant for sea water.

The benthic stage of *M. leucophaeata,* able to survive for more than 3 months at salinities of 0.2–17.5 PSU, can be transported via attachment to ship hulls in fresh or brackish water. Transport over sea can also take place as larvae in ballast water. *D. polymorpha* survived for more than 3 months in salinities of 0.2–6.0 PSU and can thus be transported via attachment to ship hulls in fresh water or as larvae or adults in ballast water. *M. edulis* survived for more than 3 months in salinities of 10.5–36.0 PSU and can be transported via attachment to ship hulls only under sea water conditions or by short trips through fresh water to marine ports.

Based on the broad tolerance limits and potential ecological plasticity, we suggest that a revision of the current accepted view on the invasion potential of these species is required based on tolerance experiments undertaken with samples from various populations. Finally, we conclude that there is a need for large-scale and long-term monitoring surveys and field transplant experiments of dreissenid and mytilid populations in water salinity gradients in estuaries and continental water bodies where they already occur, taking (local) environmental conditions into account. Future research should aim to establish physiological limits and determine to what extent the occurrence of populations is due to adaptability to fluctuations in physicochemical factors on a local or even global scale.
